# Pharmacokinetics of Oral Taurine in Healthy Volunteers

**DOI:** 10.4061/2010/346237

**Published:** 2010-06-29

**Authors:** Mohammadreza Ghandforoush-Sattari, Siminozar Mashayekhi, Channarayapatna V. Krishna, John P. Thompson, Philipp A. Routledge

**Affiliations:** ^1^NPMC, Hemmatology and Oncology Research Centre, Tabriz University of Medical Sciences, Tabriz, Iran; ^2^Tropical and Infectious Diseases Research Centre, Tabriz University of Medical Sciences, Tabriz, Iran; ^3^Faculty of Pharmacy, Tabriz University of Medical Sciences, Tabriz, Iran; ^4^Department of Pharmacology and Therapeutics, College of Medicine, Cardiff University, Heath Park, Cardiff, UK

## Abstract

Taurine, a sulfur-containing amino acid, is a normal constituent of the human diet. Little is known of the pharmacokinetics of taurine in man after oral administration. We studied the pharmacokinetics of 4 g taurine in eight healthy male volunteers (median age 27.5, range 22–45) following orally administration in the fasting state in the morning. Blood samples were taken at regular intervals and plasma taurine concentration was measured by a modified HPLC method. Data were subjected to noncompartmental analysis. Maximum plasma taurine concentration (C_max_) was measured at 1.5 ± 0.6 hr after administration as 86.1 ± 19.0 mg/L (0.69 ± 0.15 mmol). Plasma elimination half-life (T_1/2_) and the ratio of clearance/bioavailability (Cl/F) were 1.0 ± 0.3 hr and 21.1 ± 7.8 L/hr, respectively. Since taurine is occasionally used in therapeutics as a medicine, the pharmacokinetics and effects of oral taurine in healthy volunteers would be useful in the future studies of taurine in pharmacology and nutrition.

## 1. Introduction

Taurine, a sulfur-containing amino acid, is a relatively nontoxic substance and a normal constituent of the human diet [1]. The diet provides most taurine either directly or by synthesis in the liver and brain from methionine or cysteine via cysteic acid or hypotaurine [2] or via cysteamine in the heart and kidney. Taurine stabilises membranes, modulates calcium transport, and is able to dissipate the toxic effects of hypochlorous acid (HOCl) by the formation of the relatively stable taurochloramine molecule, generated by myeloperoxidases from oxygen radicals. The ability of taurine to conjugate with xenobiotics, retinoic acid, and bile salts and its role as a major free amino acid in regulating the osmolality of cells are also examples of protective functions [3]. Obinata et al. showed ALT concentrations recovering in children with fatty liver after 6-months treatment with oral taurine administered daily [4]. Protective effects of taurine against arteriosclerosis [5], lung injury by oxidant gases [6], deleterious effects of various drugs such as tauromustine, an antitumor agent, [7], hepatotoxicity of sulfolithocholate [8], and its promotion of the recovery of leukocytes in irradiated rats [9] have already been studied on animals. The therapeutic effects of taurine on epilepsy [10], ischemia [11], obesity [12], diabetes [13], hypertension [14], Congestive heart failure [15], noxious effect of smoking [16], toxicity of methotrexate [17] myocardial infarction [18], alcoholic craving [19], and neurodegeneration in elderly [20] have also been reported. Taurine may protect membranes by detoxification of destructive compounds and/or by directly preventing alterations in membrane permeability [21]. Some foods or drinks, for example, Red Bull energy drink [22], boosters, eye drops, and eardrops, contain a considerable amount of taurine [23]. Little is known of the pharmacokinetics of taurine in man after oral administration. Such information is essential if a regimen for administration of this agent to patients (e.g., after paracetamol poisoning) is designed. A literature review revealed only one report concerning the pharmacokinetics of taurine performed by Zhang et al. [24] using 200 mg IV injection form of taurine in six patients with hypertension, but the paper was brief and only available in mandarin. We therefore studied the pharmacokinetics and effects of oral taurine in healthy volunteers that would be useful in the future studies of taurine in pharmacology and nutrition

## 2. Materials and Methods

Eight healthy male volunteers (age between 22–45 year, med. 27.5 and weight between 69–122 kg, med. 79.5 kg) were recruited from the general population after fully informed written consent and after getting approval from the ethics committee of Bro Taf Health Authority of Wales, UK. Each taurine capsule contained 1000 mg (0.008 moL) taurine, manufactured by Life Extension Foundation Buyers Club, Inc (USA). Taurine 4 g (32 mmoL) was administered orally to each volunteer in the fasting state in the morning. Subjects were asked to avoid taking any proprietary medicine including prescribed or recreational drugs, eating fish and any seafood or dairy products, and drinking “Red-Bull” 24 hours before and 48 hours after the study. They were given toast and jam with a cup of tea one hour after starting the study and a normal meal without any seafood at 4 hr of the study. Blood samples were taken (3 mL each time) at regular intervals over the following times: 0, 0.5, 1, 1.5, 2, 2.5, 3, 3.5, 4, 5, 6, 7, and 8 hours and at 24 and 48 hours using cannulae in the brachial vain and collected into heparinised tubes. The samples were immediately centrifuged at 4°C at 3000 rpm. Plasma was removed using a Pasteur pipette and transferred into 5ml glass tubes and kept frozen at −20°C until analysis. Plasma taurine concentration was measured by a modified HPLC method. This method was sensitive enough, to quantify 150 pg/mL and detect 50 pg/mL of taurine ranging normally between 65 and 179 mmol/L (8–22 *μ*g/mL) [25]. The pharmacokinetic parameters of area under the concentration curve (AUC_0–8 h_), maximum concentration (C_max⁡_), time of C_max⁡_ (T_max⁡_), plasma half-life (T_1/2_), volume of distribution (V), and the ratio of clearance/bioavailability (Cl/F) were calculated using WinNonlin (Version 1.5) software packages. The data were used to develop a noncompartmental pharmacokinetic model, which might be suitable for patient studies in the future. Since plasma taurine concentration returned to endogenous level after 8 hr of study, the data after 8 hr were ruled out of the pharmacokinetic analysis. In addition, since this was a study of kinetics of exogenously administered taurine, baseline endogenous concentrations of taurine in plasma (0.04 ± 0.0 mmoL) were also excluded from the study. Therefore, the changes in plasma taurine concentration from baseline were calculated.

## 3. Results

**Figure 1:**
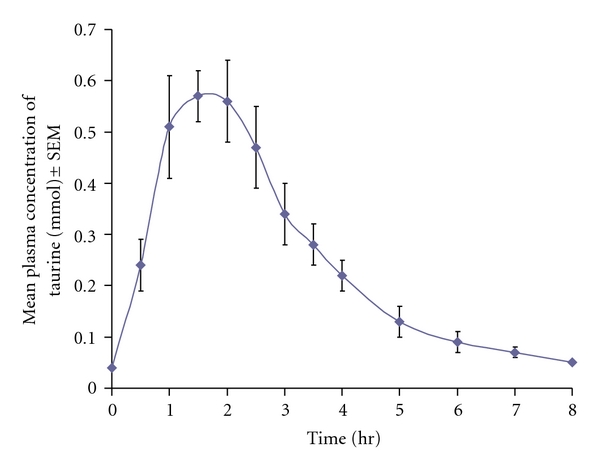
Linear plot of mean plasma taurine levels (mmoL) in eight healthy volunteers following administration of 4 g (32 mmoL) oral taurine.

Plasma taurine values from 0–48 hr in eight healthy volunteers after administration of 4 g taurine capsules are listed in Table 1. Data showed that endogenous plasma taurine concentrations before taking the taurine capsules ranged from 0.03 to 0.06 mmoL (mean 0.04 ± 0.0 mmoL). Time to reach maximum concentration ranged from 1 to 2.5 hr (mean 1.5 ± 0.6 hr) (absorption phase). The mean maximum plasma taurine concentration was 0.57 ± 0.05 mmoL. Plasma taurine concentrations returned to normal range at 8 hr (elimination phase) (Figure 1). 

Mean changes in plasma taurine concentrations from baseline showed that the absorption phase for taurine after oral administration of 4 g taurine capsules took 1.5 hr to reach the peak concentration (0.53 ± 0.1 mmoL) and then returned to normal range (0.04 ± 0.0 mmoL) in 6.5 hr (Figure 1). The pharmacokinetic parameters of taurine after oral administration of 4 g taurine capsules are shown in Table 2. Plasma taurine concentration peaked to 59.0–112.6 mg/L (mean 86.1 ± 19.0) at 1–2.5 hr of study, plasma elimination half-life ranged from 0.7 to 1.4 hr (mean 1.0 ± 0.3), volume of distribution ranged from 19.8 to 40.7 L (mean 30.0 ± 7.6), ratio of clearance/bioavailability (Cl/F) ranged from 14.0 to 34.4 L/hr (mean 21.1 ± 7.8), and area under curve between 0–8 hr (AUC) ranged from 116.0 to 284.5 mg·hr/L (mean 206.3 ± 63.9).

## 4. Discussion

**Figure 2:**
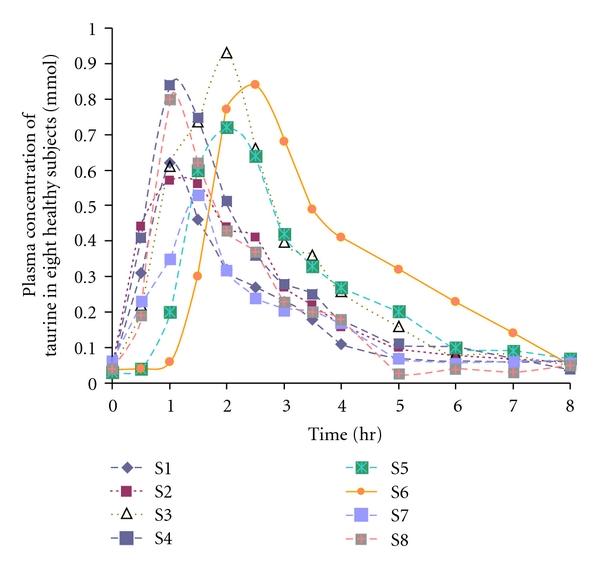
Changes in plasma taurine (mmoL) from baseline in eight healthy volunteers following administration of 4 g (32 mmoL) oral taurine.

Taurine has already been used intravenously in humans in doses of up to 5 g [26] and 2–6 g/day orally for a period of 6 months in children with fatty liver [4] without any toxic side effect. In the human adult, about one-fourth of bile acids are conjugated with taurine and a small fraction of taurine is also converted to isethionate by either bacterial or tissue enzymes and may be converted in part to sulphate, CO_2_, water, and ammonia, the last being converted to urea [27]. Total body taurine is regulated by the kidney. Taurine is a major urinary amino acid in humans because the capacity of renal uptake is low [2, 28]. Daily taurine losses in urine are diet-dependent but generally range from 65 to 250 mg (0.5–2.0 mmoL) [27]. With few exceptions, animal [2] and human [15] studies have demonstrated that taurine, even in high doses, is generally free of any serious adverse effects. In the present study, no significant change in the systolic or diastolic blood pressure and pulse rate was observed during the study and the volunteers had no complaint during the study. In the present study, data showed that oral taurine was absorbed from the gastrointestinal tract 1–2.5 hr following administration and then eliminated from plasma by first order kinetics. Even though the volunteers had been asked to avoid eating anything before coming to the trial, two subjects (3 and 6), whose absorption phase took 2 and 2.5 hr, respectively, may not have taken the drug with an empty stomach (Figure 2). 

Plasma taurine returned to endogenous concentrations after 6–8 hr of study. Therefore, there was no need to follow up the drug in plasma after 8 hr. 

A literature review revealed only one report concerning the pharmacokinetics of taurine [24]. Zhang et al. studied the pharmacokinetics of an IV injection of a 200 mg bolus dose on six hypertensive human patients and six healthy volunteers. Plasma half-life and volume of distribution of taurine in Zhang et al.'s was 3.85 ± 0.05 min and 9.6 ± 3.2 L, respectively. However, they only followed the plasma taurine concentrations for 20 min, and therefore, they were probably examining an alpha phase which was obscured by the absorption phase for taurine after oral absorption. Further studies are necessary to elucidate the optimum dose of oral taurine for protecting cells against toxic agents in human.

## Figures and Tables

**Table 1:** Plasma taurine concentrations (mmoL) in eight healthy volunteers after administration of 4 g (32 mmoL) oral taurine.

Time (hr)	Subjects								Mean	SEM
	1	2	3	4	5	6	7	8		
0	0.06	0.04	0.03	0.03	0.03	0.04	0.06	0.04	0.04	0.00
0.5	0.31	0.44	0.22	0.41	0.04	0.04	0.23	0.19	0.24	0.05
1	0.62	0.57	0.61	0.84	0.20	0.06	0.35	0.80	0.51	0.10
1.5	0.46	0.56	0.74	0.75	0.60	0.30	0.53	0.62	0.57	0.05
2	0.32	0.44	0.93	0.51	0.72	0.77	0.32	0.43	0.56	0.08
2.5	0.27	0.41	0.66	0.36	0.64	0.84	0.24	0.37	0.47	0.08
3	0.23	0.27	0.40	0.28	0.42	0.68	0.21	0.23	0.34	0.06
3.5	0.18	0.22	0.36	0.25	0.33	0.49	0.20	0.20	0.28	0.04
4	0.11	0.16	0.26	0.18	0.27	0.41	0.17	0.18	0.22	0.03
5	0.07	0.10	0.16	0.11	0.20	0.32	0.07	0.03	0.13	0.03
6	0.06	0.08	0.08	0.10	0.10	0.23	0.06	0.04	0.09	0.02
7	0.06	0.07	0.08	0.07	0.09	0.14	0.06	0.03	0.07	0.01
8	0.06	0.06	0.05	0.04	0.07	0.05	0.06	0.05	0.05	0.00
24	0.06	0.04	0.03	0.02	0.04	0.06	0.07	0.06	0.05	0.01
48	0.05	0.04	0.04	0.02	0.02	0.06	0.09	0.04	0.05	0.01

**Table 2:** Pharmacokinetic parameters of taurine after oral administration of 4 g (32 mmoL) taurine capsules.

Subjects	C_max⁡_ (mg/L)	T_max⁡_ (hr)	AUC_(0-8 hr)_ (mg·hr/L)	K_*e*_ (hr^−1^)	T_1/2 _(hr)	V (L)	Cl/F (L/hr)
1	69.7	1	127.7	0.8	0.8	37.8	31.2
2	66.7	1	198.0	0.5	1.4	40.7	19.5
3	112.6	2	281.4	0.6	1.1	22.1	14.0
4	100.4	1	235.0	0.6	1.1	26.8	16.9
5	86.1	2	232.0	0.5	1.4	32.5	16.6
6	99.5	2.5	284.5	0.7	1	19.8	14.0
7	59.0	1.5	116.0	1.0	0.7	34.8	34.4
8	94.8	1.0	175.9	0.9	0.8	25.1	22.2

Mean	86.1	1.5	206.3	0.7	1.0	30.0	21.1
SD	19.0	0.6	63.9	0.2	0.3	7.6	7.8
Range	59.0–112.6	1–2.5	116.0–284.5	0.5–1.0	0.7–1.4	19.8–40.7	14.0–34.4
